# The Role of Testosterone in Spermatogenesis: Lessons From Proteome Profiling of Human Spermatozoa in Testosterone Deficiency

**DOI:** 10.3389/fendo.2022.852661

**Published:** 2022-05-19

**Authors:** Giuseppe Grande, Ferran Barrachina, Ada Soler-Ventura, Meritxell Jodar, Francesca Mancini, Riccardo Marana, Sabrina Chiloiro, Alfredo Pontecorvi, Rafael Oliva, Domenico Milardi

**Affiliations:** ^1^ Research Group on Human Fertility, International Scientific Institute “Paul VI”, Rome, Italy; ^2^ Division of Endocrinology, Fondazione Policlinico Universitario “Agostino Gemelli” Scientific Hospitalization and Treatment Institute (IRCCS), Rome, Italy; ^3^ Department of Biomedical Sciences, Molecular Biology of Reproduction and Development Research Group, Institut d’Investigacions Biomèdiques August Pi i Sunyer (IDIBAPS), Fundació Clínic per a la Recerca Biomèdica, Faculty of Medicine and Health Sciences, University of Barcelona, Barcelona, Spain; ^4^ Biochemistry and Molecular Genetics Service, Hospital Clínic, Barcelona, Spain; ^5^ Department of Translational Medicine and Surgery, Catholic University of the Sacred Heart, Rome, Italy

**Keywords:** sperm, hypogonadism, proteomics, LH deficiency, testosterone

## Abstract

Testosterone is essential to maintain qualitative spermatogenesis. Nonetheless, no studies have been yet performed in humans to analyze the testosterone-mediated expression of sperm proteins and their importance in reproduction. Thus, this study aimed to identify sperm protein alterations in male hypogonadism using proteomic profiling. We have performed a comparative proteomic analysis comparing sperm from fertile controls (a pool of 5 normogonadic normozoospermic fertile men) versus sperm from patients with secondary hypogonadism (a pool of 5 oligozoospermic hypogonadic patients due to isolated LH deficiency). Sperm protein composition was analyzed, after peptide labelling with Isobaric Tags, *via* liquid chromatography followed by tandem mass spectrometry (LC-MS/MS) on an LTQ Velos-Orbitrap mass spectrometer. LC-MS/MS data were analyzed using Proteome Discoverer. Criteria used to accept protein identification included a false discovery rate (FDR) of 1% and at least 1 peptide match per protein. Up to 986 proteins were identified and, of those, 43 proteins were differentially expressed: 32 proteins were under-expressed and 11 were over-expressed in the pool of hypogonadic patients compared to the controls. Bioinformatic analyses were performed using UniProt Knowledgebase, and the Gene Ontology Consortium database based on PANTHER. Notably, 13 of these 43 differentially expressed proteins have been previously reported to be related to sperm function and spermatogenesis. Western blot analyses for A-Kinase Anchoring Protein 3 (AKAP3) and the Prolactin Inducible Protein (PIP) were used to confirm the proteomics data. In summary, a high-resolution mass spectrometry-based proteomic approach was used for the first time to describe alterations of the sperm proteome in secondary male hypogonadism. Some of the differential sperm proteins described in this study, which include Prosaposin, SMOC-1, SERPINA5, SPANXB1, GSG1, ELSPBP1, fibronectin, 5-oxoprolinase, AKAP3, AKAP4, HYDIN, ROPN1B, ß-Microseminoprotein and Protein S100-A8, could represent new targets for the design of infertility treatments due to androgen deficiency.

## Introduction

Testosterone (T) is the androgen in the testis that is required for initiating and maintaining spermatogenesis, and the production of mature sperm is intimately dependent on androgen action within the testis. Therefore, in the scenario of an absence of T, or its receptor, spermatogenesis does not proceed beyond the meiosis stage and results in male infertility (1). In men, Leydig cells are the responsible for producing T after being stimulated by luteinizing hormone (LH), a glycoprotein hormone secreted from the pituitary gland in response to the pulsatile release of gonadotropin-releasing hormone (GnRH) from the hypothalamus (2).

Male hypogonadism is a clinical syndrome that results from the failure of the testis to produce physiological concentrations of T and/or a normal number of spermatozoa due to pathology at one or more levels of the hypothalamic–pituitary–testicular axis (3). For example, an impairment of the pituitary or hypothalamic function can lead to LH deficiency, which is a form of secondary hypogonadism. Secondary hypogonadism, also named hypogonadotropic hypogonadism, is characterized by low T concentrations in serum, reduced spermatogenesis, and inappropriately low concentrations of gonadotrophins (LH and follicular stimulating hormone (FSH)) (4). LH deficiency usually occurs in conjunction with FSH deficiency. However, isolated LH deficiency is a rare clinical condition, originating from a dysregulation in the hypothalamus-pituitary GnRH-LH-testosterone axis, which results in an isolated dysregulation of LH secretion, with a normal or low-normal FSH secretion (5). Both pituitary gonadotrophins LH and FSH, together with a high LH-stimulated intratesticular T concentration, are considered crucial for spermatogenesis and are required for quantitatively normal sperm production (6). FSH is particularly important for establishing a normal and functional Sertoli cell population, LH for promoting the production of T, whereas androgen action is needed for the fulfillment of germ cell development. The stimulation of spermatogenesis by androgen needs a direct action on androgen receptors (AR) in Sertoli cells. T is, in fact, essential for maintaining qualitative spermatogenesis (2) and it is required for at least four critical processes: maintenance of the blood-testis barrier (BTB) (7), meiosis (8), Sertoli-spermatid adhesion (9), and sperm release (10). Although T is essential to maintain good spermatogenesis, a study performed in genetically modified mice displaying a strong FSH stimulation, together with a minimal T production, showed near-normal spermatogenesis (11). Thus, this result reflects that a strong FSH signaling can maintain spermatogenesis also in presence of low levels of intratesticular T.

The molecular mechanisms of T action in spermatogenesis have not been completely revealed until recently. Mice lacking Sertoli cell androgen receptors (AR) show late meiotic germ cell arrest, suggesting Sertoli cells transduce the androgenic stimulus coordinating this essential step in spermatogenesis. Specifically, the loss of T was found to alter the expression and post-translational modifications of meiotic cells proteins involved in oxidative damage, DNA repair, RNA processing, apoptosis, and meiotic division (12).

With the advances obtained using high-throughput techniques, such as proteomics, we may now target the role of T in spermatogenesis. Proteomics represents a state-of-the-art, technology-driven science, which allows studying, in a high-throughput mode, proteins, protein modifications, and protein interactions. This powerful tool is currently widely used to elucidate complex biological processes, including fertility and infertility (13, 14). Therefore, proteomics might represent a novel platform for clinical research to investigate the *in vivo* effect of hormones on the protein expression of cells, tissues, and biological fluids. However, in humans, no studies have been yet performed to confirm the T-mediated expression of sperm proteins and their functional importance.

Therefore, to better understand the impact of T in the sperm proteome profile, the aim of this study is to evaluate human sperm protein composition by high-resolution mass spectrometry in patients with male hypogonadism displaying low intratesticular T as a condition reported in isolated LH deficiency.

## Materials and Methods

### Experimental Rationale

The rationale for studying only men with secondary hypogonadism due to isolated LH deficiency was to select a condition of a severe reduction in both blood and intratesticular T levels, without other confounding risk factors. Primary hypogonadal patients were excluded because of their high LH levels, which can represent a confounding factor, since it may maintain minimal intratesticular T levels, although blood T levels are reduced. Furthermore, patients with primary hypogonadism often show normal or increased serum estrogen levels, which might represent a confounding factor. On the other side, patients with secondary hypogonadism and reduced values of both FSH and LH values have generally azoospermia since low FSH is associated with an impairment in spermatogenesis. We, therefore, selected a rare clinical model (secondary hypogonadism due to isolated LH deficiency), in which we may observe LH and testosterone deficiency associated with a normal or low-normal FSH secretion, and presence of spermatogenesis.

### Subjects

Six male hypogonadic patients aged between 25- and 55-year-old with secondary hypogonadism due to isolated LH deficiency were consecutively enrolled for this study. The diagnosis of secondary hypogonadism due to LH deficiency was done in presence of symptoms of male hypogonadism (e.g.: loss of body hair, reduced sexual desire and activity, decreased spontaneous erections, erectile dysfunction, and gynecomastia) and low levels of blood T and LH (3), which were further confirmed by the GnRH test (15). A pituitary magnetic resonance imaging (MRI) was performed in all patients to confirm the diagnosis of pituitary disease. Inclusion criteria were as follows (1): total T < 2.5 ng/ml (2), LH < 1.0 mUI/ml, and (3) clinical symptoms of hypogonadism. Exclusion criteria included (1): age <18 yrs and >55 yrs (2), primary hypogonadism or associated testicular diseases (3), residual adenoma (4), smoking (5), diabetes mellitus (6), previous androgen replacement therapy (7), varicocele, and/or (8) genital tract infections.

In addition, five normogonadic normozoospermic fertile men, whose partners were pregnant when the study started, were enrolled as a control group. None had a history of fertility problems. All female partners conceived within 3 months before the start of the study.

### Hormonal Study

A blood sample was collected at 08.00 hours in the andrological clinic of Fondazione Policlinico “A. Gemelli” IRCCS in Rome (Italy), for the determination of T, estradiol (E2), sex hormone–binding globulin (SHBG), LH, and FSH. T and E2 were assayed in duplicate by radioimmunoassay (RIA) with the use of commercial kits by Radim (Pomezia, Italy). LH, FSH, and SHBG were assayed by immunoradiometric methods on a solid-phase (coated tube), which is based on a monoclonal double-antibody technique. The reference values of the studied hormones are reported in **Table 1**. Results have been reported as average ± standard deviation. Statistical analysis has been carried out with SPSS v18.0 (IBM Corp., Armonk, NY, USA). All data have been first analyzed for normality of distribution using the Kolmogorov–Smirnov test of Normality. The appropriate parametric test (t-test) was used to assess the significance of the differences between groups. P-value < 0.05 was considered significant.

**Table 1:** Clinical, hormonal and seminal parameters in secondary hypogonadism patients and normogonadic controls.

	Hypogonadic patients (n=5)	Fertile controls (n=5)	Range values/Lower reference limit
Testosterone (T)	1.93 ± 0.31*	5.2 ± 0.8	2.5-8.4 ng/ml
Estradiol (E2)	26.92 ± 5.16	26.8 ± 9.3	15-44 pg/ml
FSH	2.14 ± 0.70	2.5 ± 1.2	1.0-8.0 mU/ml
LH	0.81 ± 0.12*	2.5 ± 0.9	2.5-10.0 mU/ml
Seminal volume	2.20 ± 1.15 ml*	4.10 ± 1.85 ml	1.5 ml
Total sperm count	21.00 ± 10.19 x 10^6^*	98.60 ± 49.94 x 10^6^	39 x 10^6^
Total sperm motility	59.00 ± 12.45%	63.0 ± 19.87%	40%
Normal morphology	3.10 ± 2.4%*	21.2± 7.91%	4%

P-value < 0.05 is indicated in the table (*).

The diagnosis of male secondary hypogonadism was moreover confirmed by the GnRH test (100 μg i.v., LHRH, Ferring Pharmaceuticals, Saint-Prex, Switzerland) for FSH and LH hormones. Additionally, the hormonal diagnosis of secondary hypogonadism was further corroborated by pituitary MRI performed in all patients.

### Semen Sample Collection and Analysis

Human semen samples were obtained from 6 male patients diagnosed with secondary hypogonadism due to LH deficiency and 5 fertile controls at the andrological clinic of Fondazione Policlinico “A. Gemelli” in Rome (Italy). All subjects gave informed consent according to the guidelines of the Declaration of Helsinki. Complete semen analysis for all individuals was performed according to World Health Organization (2010) classification (16). Only seminal samples from secondary hypogonadism individuals with sperm presence were used for the current study.

### Sperm Preparation and Purification

The ejaculates were washed with PBS and the sperm cells were selected after 50% Percoll™ gradient (GE Healthcare, Uppsala, Sweden), as previously described (17, 18). Briefly, the sperm samples were centrifuged through a 50% Percoll gradient at 400 g for 30 min at room temperature (RT) without brake. The recovered sperm cells were then resuspended in phosphate-buffered saline (PBS; Sigma-Aldrich, St. Louis, MO, USA) and subjected to a residual leukocyte depletion using Dynabeads™ CD45 (Invitrogen, Carlsbad, CA, USA). Basically, 1 ml aliquots of samples were incubated with 50 µl washed dynabeads for 1 hour at RT, with constant shaking. Samples were washed twice by applying magnetic force for 2 min, and the efficiency of the procedure was checked using phase-contrast microscopy (Olympus BX50, Olympus, Tokyo, Japan).

### Quality Control to Assess Sperm Contamination

The absence of leukocytes was further confirmed both by microscopic observation after Diff-Quick staining and by performing a real-time PCR for the leukocyte-specific marker Protein Tyrosine Phosphatase Receptor Type C (PTPRC). For Diff-Quick staining, sperm smears were prepared by placing 5 µl of the sample onto a slide and pulling it out into a smear using a second slide followed by air drying for 20–30 s. The staining kit used was Diff-Quick (Medion Diagnostics AG, Düdingen, Switzerland), and smears were stained according to the manufacturer’s instructions.

For sperm RNA analysis, sperm RNA was individually extracted and purified following the RNeasy Mini Kit protocol (Qiagen, Hilden, Germany) with some modifications previously established (19). The possible somatic contamination from leukocytes was further assessed by reverse transcription reaction using SuperScript III RT (Invitrogen, Carlsbad, CA, USA) and oligodT primers (Invitrogen, Carlsbad, CA, USA), and a subsequent real-time PCR using PowerUp™ SYBR™ Green PCR (Applied Biosystems, Foster City, CA, USA) targeting the leukocyte-specific marker Protein Tyrosine Phosphatase Receptor Type C (PTPRC). The absence of amplification of the leukocyte-specific marker *PTPRC* PCR product at cycle 40 in sperm RNA isolated from the individual samples included in the proteomics study confirmed the absence of leukocyte RNA contamination (**Supplementary Table 1**). Likewise, to assess sample RNA integrity, protamine 1 (*PRM1*) was targeted as a positive control of a sperm-specific intact RNA (**Supplementary Table 1**).

In conclusion, only sperm samples with no visible contamination with other cells and negative for *P*TPRC at the mRNA level were used for further proteomic analysis. Consequently, one sample from an individual with secondary hypogonadism was discarded due to leukocyte contamination at both microscopic observation and specific RNA-leukocyte analysis.

### Protein Solubilization

Protein solubilization was independently performed on each sperm sample. Briefly, after sperm purification, the sperm pellet was solubilized in a 2% SDS lysis buffer. Lysates were centrifuged at 16,000 g for 10 min, and the proteins present in the supernatant were quantified using Pierce™ BCA Protein Assay Kit (Thermo Fisher Scientific, Rockford, IL, USA).

### Differential Proteomics

A comparative sperm proteomic analysis was performed comparing two pools: a fertile control pool (n=5) versus a secondary hypogonadic patients pool (n=5). Due to the low sperm count and limited material in the patients diagnosed with secondary hypogonadism, a sample pooling strategy was considered for the current study, despite the potential limitations of this approach.

To prepare the two pools, 25 µg of each sample were used (125 µg of total protein for each pool). The TMTduplex™ Isobaric Mass Tagging Kit (TMT 2-plex; Thermo Fisher Scientific, Rockford, IL, USA) was used for the peptide labeling, following the manufacturer’s instructions with minor modifications (13).

### Peptide Labeling With Isobaric Tags (TMT 2-Plex)

For the preparation of the TMT labeling, 80 µg of proteins from each pool were suspended in 100 mM TEAB (triethyl ammonium bicarbonate, pH 8.5) and the same concentration (0.85 µg/µl) and volume were acquired for both pools. Proteins were reduced in 9.5 mM TCEP (tris (2-carboxyethyl) phosphine) for one hour at 55 °C. Then, proteins were alkylated with 17 mM iodoacetamide for 30 min at RT avoiding light exposure. Proteins were precipitated overnight at -20°C with 80% (v/v) cold acetone. Afterward, samples were centrifuged at 17,500 g for 10 min at 4°C, and the acetone-precipitated protein pellets were resuspended in 80 µl of 100 mM TEAB. For protein digestion, trypsin was added to the protein pellets at a 1:20 protease-to-protein ratio and incubated overnight at 37°C with constant shaking. Regarding peptide labeling, 30 µg of peptides from each pool were labeled with TMT isobaric tags (the control group was labeled with TMT-126, and the secondary hypogonadic patients with TMT-127). Specifically, TMT label reagents (0.8 mg each) were equilibrated at RT, dissolved in 41 µl of anhydrous acetonitrile (ACN; Sigma-Aldrich, St. Louis, MO, USA), and added to the reduced and alkylated peptides of each pool. After 1 hour of incubation at RT, the reaction was quenched with 8 µl of 0.5% hydroxylamine for 15 min at RT under shaking. Then, the TMT-labeled pools were combined at equal amounts in a single tube. After, labeled peptides were dried in a vacuum centrifuge and re-suspended in 20 µl of 0.5% trifluoroacetic acid (TFA; Sigma-Aldrich, St. Louis, MO, USA) in 5% ACN before processing with Pierce C18 Spin Columns (Thermo Scientific, Rockford, IL, USA) following manufacturer’s instructions.

### LC-MS/MS

The dried peptides pellet was reconstituted in 0.1% formic acid and injected for analysis by reverse-phase chromatography-MS/MS (Tandem mass spectrometry). Specifically, tryptic peptides were separated by using a reversed-phase nano LC-MS/MS setup comprised of nano-LC Ultra 2D Eksigent (AB Sciex, Brugg, Switzerland) attached to an LTQ-Orbitrap Velos mass spectrometer (Thermo scientific, San Jose, CA, USA). Peptides were loaded onto a C18 trap column (5 µm, 120 Å, 100 µm i.d. x 2 cm in length, Nanoseparations). For analytical separation, a gradient was applied on line with an analytical column (3 µm, 100 Å, 75 µm i.d. x 15 cm in length, Thermo Scientific, San Jose, CA, USA), and the following buffer system was used: buffer A (97% H2O-3% ACN, 0.1% Formic acid) and buffer B (3% H2O-97% ACN, 0.1% Formic acid). For the peptide mixtures the following gradient was applied: 0–5 min 0% to 5% B, 5–285 min 5% to 40% B, 285-290 min 40% to 100% B, 290-300 min 100% to 100% B at a flow rate of 400 nl/min. MS/MS analysis was performed using an LTQ-Orbitrap Velos (Thermo Fisher Scientific, Waltham, MA, USA) with a nanoelectrospray ion source with precursor ion selection in the Orbitrap at 30,000 of resolution, in a range of 400-1700 m/z, selecting the 15 most intense precursor ions in positive ion mode. MS/MS data acquisition was completed using Xcalibur 2.1 (Thermo Fisher Scientific, Waltham, MA, USA). Higher energy collisional dissociation (HCD) for MS2 was set to 40%.

### Database Searching and Data Interpretation

Data were processed using Proteome Discoverer 1.4.1.14 (Thermo Fisher Scientific, Waltham, MA, USA). For database searching, processed data were submitted to the Homo sapiens UniProtKB/Swiss-Prot database with Sus scrufa Trypsin (HUMAN_PIG_UniProt_R_2017_01.fasta; released January 2017; 21,484 protein entries) added to it using SEQUEST, version 28.0 (Thermo Fisher Scientific, Waltham, MA, USA). The percolator search node was used for re-scoring. The following search parameters were used: five maximum missed cleavages for trypsin, TMT-labeled lysine in N-terminal (+225.156 Da) and methionine oxidation (+15.995 Da) as dynamic modifications, cysteine carbamidomethylation (+57.021 Da) as a static modification, 20 ppm precursor mass tolerance, 0.1 Da fragment mass tolerance, and 100 mmu peak integration tolerance was applied, and most confident centroid peak integration method was used.

Criteria used to accept protein identification included a false discovery rate (FDR) of 1% and at least 1 peptide match per protein. In addition, proteins have been treated as “ungrouped” to avoid any possible ambiguity among the different isoforms of the same protein. The relative quantification of proteins was achieved by dividing the intensity of reporter ions of HCD MS2 spectra for secondary hypogonadism patients pool (TMT-127) with the controls pool (TMT-126), which were obtained using Proteome Discoverer software. Purity correction factors provided by the manufacturer were applied to the isotopic purities of the reporter ions.

For protein quantification purposes, only unique peptides were used, and protein ratios (TMT-127/TMT-126) were normalized to the protein median. The normalized protein ratios were log2 transformed and the 95% confidence interval was calculated (mean ± 1.96 SD), and the protein ratios outside the range were defined as significantly different (p-value ≤ 0.05). Log2 values were reverted to normal values, and the cut-off for up-regulated proteins was ≥1.264, and for down-regulated proteins was ≤ 0.768.

Proteins differentially expressed in hypogonadic patients and controls were classified according to their main cellular function(s) using the information available at the UniProtKB/Swiss-Prot database (http://www.uniprot.org). The sperm proteomic datasets were uploaded to the Gene Ontology Consortium database (http://www.geneontology.org/) (20), based on PANTHER v17 database (Release date 2021-10-01), to predict the functional involvement of the deregulated sperm proteins in male hypogonadism. The significance of enrichment analyses was calculated by a Fisher’s exact test. P-values < 0.05 after FDR adjustment were considered statistically significant.

### Western Blot

In order to validate the proteomics results, western blot analyses were performed in protein extracts of 4 hypogonadic sperm samples used for the proteomics study, and 4 controls. For western blot analysis, sperm cells were diluted in 2% SDS lysis buffer. The protein concentrations were measured using a BCA protein assay. Samples were resuspended in SDS Laemmli sample buffer and boiled for 5 min at 95°C. SDS–PAGE was transferred onto PVDF membranes (Millipore, Burlington, MA, USA). Membranes were developed using enhanced chemiluminescence (ECL Amersham - GE Healthcare, Little Chalfont, UK). For immunostaining, the anti-AKAP3 rabbit monoclonal antibody (Dilution 1:1000; Abcam, Cambridge, UK) and the anti-PIP rabbit monoclonal antibody (Dilution 1:1500; Novus Biologicals, Minneapolis, MN, USA) were used. The constitutively expressed tubulin protein [mouse anti-Tubulin monoclonal antibody (Dilution 1:5000; Sigma-Aldrich, Saint Louis, Missouri, US)] was used as loading controls for quantitative western blotting.

## Results

### Clinical and Seminal Parameters

The results for clinical, hormonal, and seminal data are reported in **Table 1**. Total T, LH, sperm count and sperm morphology resulted significantly reduced in the hypogonadic patients, compared to the control group (p-value < 0.05). Thus, all patients with hypogonadism enrolled in this study were oligozoospermic. The analysis of seminal parameters in those patients confirmed that spermatogenesis was conserved because of FSH levels, since sperm could be observed in the ejaculate.

GnRH test confirmed the diagnosis of LH deficiency in the individuals with hypogonadism. Pituitary MRI demonstrated the presence of partial empty sella in all patients. Empty sella syndrome is a condition in which the pituitary gland shrinks or gets flattened. Partial empty sella is suggestive that, to some extent, the pituitary gland is still visible on the MRI scan (21), as observed in our hypogonadic patients.

Therefore, the comparative proteomic study included 5 oligozoospermic hypogonadic patients and 5 normogonadic normozoospermic fertile controls. All subjects had a sperm count >10 x 10^6^ sperm/ml and leukocyte contamination was excluded at both microscopic and RNA levels.

### Differentially Abundance of Sperm Proteins in Hypogonadic Patients

LC-MS/MS of the 2 pools of sperm samples, comprising both the hypogonadic patients and the fertile control individuals, resulted in the identification of a total of 986 proteins. The comparison of the sperm proteomic profiles from hypogonadic patients and controls resulted in the detection of 43 differentially abundant proteins. Of those, 32 proteins were observed to be under-regulated, and 11 up-regulated, in the pool of hypogonadic patients, compared to controls (**Table 2**).

**Table 2:** List of up-regulated and down-regulated sperm proteins in male hypogonadism (n=32 proteins down-regulated; n=11 proteins up-regulated).

Accession	Gene name	Description	Peptide count	Unique Peptides	Ratio Hypo/Ctl
Down-regulated proteins in male hypogonadism sperm samples (n=32)					
Q9Y272	RASD1	Dexamethasone-induced Ras-related protein 1	1	1	0.425
P12273	PIP	Prolactin-inducible protein	3	3	0.514
P05164	MPO	Myeloperoxidase	1	1	0.532
P02768	ALB	Serum albumin	15	11	0.579
P05109	S100A8	Protein S100-A8	1	1	0.597
Q08380	LGALS3BP	Galectin-3-binding protein	1	1	0.604
Q9UBC9	SPRR3	Small proline-rich protein 3	1	1	0.632
P05154	SERPINA5	Plasma serine protease inhibitor	1	1	0.633
O14607	UTY	Histone demethylase UTY	1	1	0.640
Q02383	SEMG2	Semenogelin-2	22	18	0.644
P08118	MSMB	Beta-microseminoprotein	1	1	0.647
P07288	KLK3	Prostate-specific antigen	1	1	0.647
P25311	AZGP1	Zinc-alpha-2-glycoprotein	2	2	0.656
Q15843	NEDD8	NEDD8	1	1	0.663
P04279	SEMG1	Semenogelin-1	18	14	0.664
Q8TCT9	HM13	Minor histocompatibility antigen H13	1	1	0.666
Q99963	SH3GL3	Endophilin-A3	1	1	0.688
O15127	SCAMP2	Secretory carrier-associated membrane protein 2	1	1	0.702
Q6W4X9	MUC6	Mucin-6	1	1	0.705
Q9Y5C1	ANGPTL3	Angiopoietin-related protein 3	1	1	0.705
Q4G0P3	HYDIN	Hydrocephalus-inducing protein homolog	1	1	0.709
Q5JQC9	AKAP4	A-kinase anchor protein 4	34	33	0.710
P07602	PSAP	Prosaposin	2	2	0.711
A4D1T9	PRSS37	Probable inactive serine protease 37	2	2	0.724
Q4W5G0	TIGD2	Tigger transposable element-derived protein 2	1	1	0.733
Q9UIA9	XPO7	Exportin-7	2	2	0.733
O75969	AKAP3	A-kinase anchor protein 3	25	25	0.742
Q9H4F8	SMOC1	SPARC-related modular calcium-binding protein 1	1	1	0.745
Q0VFZ6	CCDC173	Coiled-coil domain-containing protein 173	1	1	0.750
Q9BTW9	TBCD	Tubulin-specific chaperone D	1	1	0.750
Q9NZL4	HSPBP1	Hsp70-binding protein 1	1	1	0.759
Q9BZX4	ROPN1B	Ropporin-1B	6	1	0.768
Up-regulated proteins in male hypogonadism sperm samples (n=11)					
P02751	FN1	Fibronectin	13	13	1.272
Q7Z5L4	SPATA19	Spermatogenesis-associated protein 19, mitochondrial	1	1	1.280
Q12765	SCRN1	Secernin-1	1	1	1.283
O14841	OPLAH	5-oxoprolinase	1	1	1.289
P62750	RPL23A	60S ribosomal protein L23a	1	1	1.296
Q9NS25	SPANXB1	Sperm protein associated with the nucleus on the X chromosome B1	4	3	1.386
P49901	SMCP	Sperm mitochondrial-associated cysteine-rich protein	2	2	1.387
Q96BH3	ELSPBP1	Epididymal sperm-binding protein 1	2	2	1.468
Q2KHT4	GSG1	Germ cell-specific gene 1 protein	1	1	1.656
Q17RY6	LY6K	Lymphocyte antigen 6K	1	1	1.672
Q9H3G5	CPVL	Probable serine carboxypeptidase CPVL	1	1	1.987

The criteria used to accept protein identification included at least 1 unique peptide and an FDR of 1%. Results are expressed as the protein ratio of sperm proteins from secondary hypogonadism patients (Hypo) to controls (Ctl).

**Figure 1:**
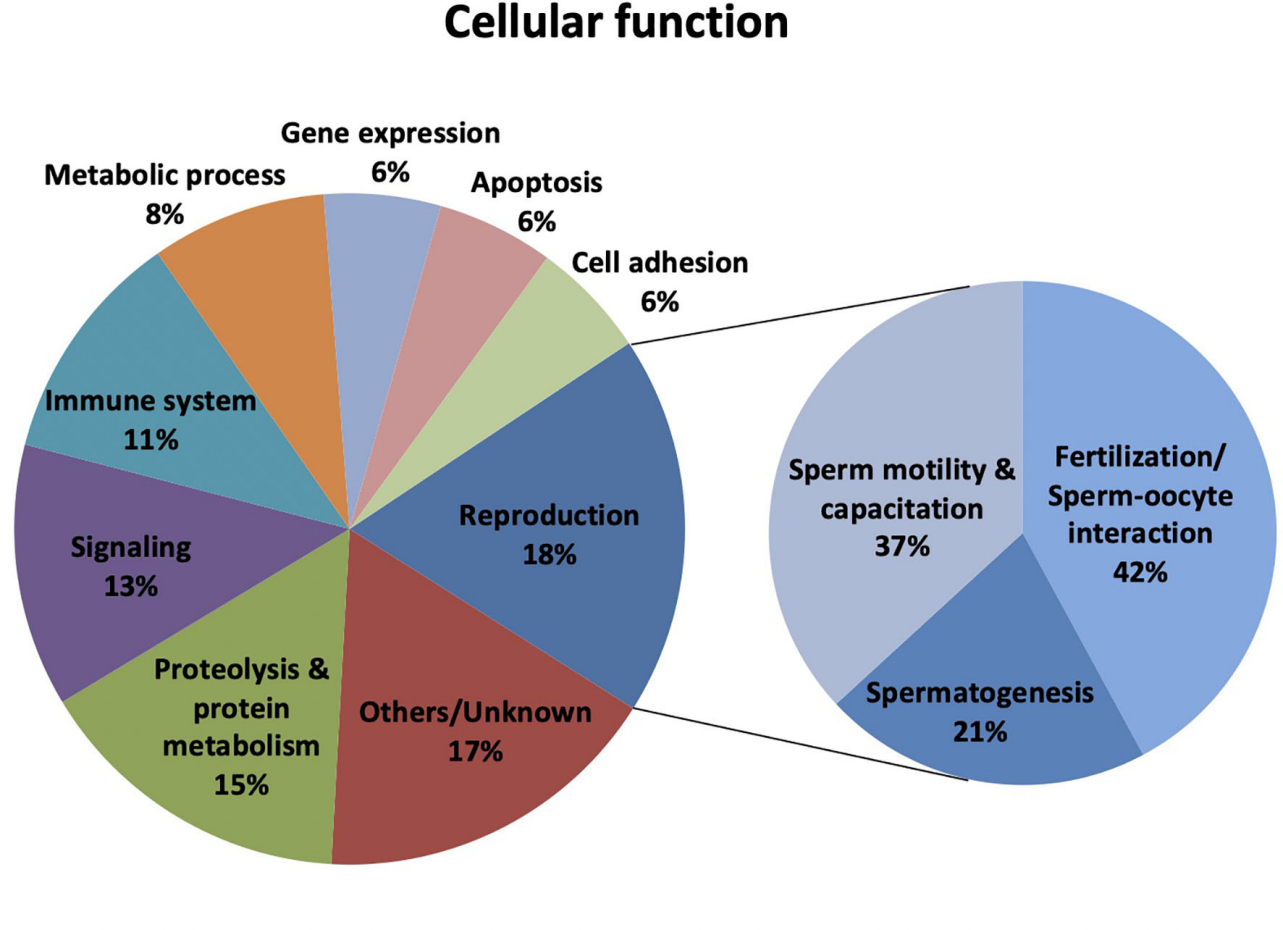
Classification of the differentially expressed sperm proteins in male hypogonadism (n=43) according to their Cellular function. Protein classification was made according to UniProtKB/Swiss-Prot database (http://www.uniprot.org).

The categorization of proteins with altered abundance in hypogonadic patients according to the information available at the Uniprot Knowledgebase revealed that 13 proteins were related to reproduction (18% of the total) (**Figure 1**). Worth mentioning, some of these proteins had more than one main cellular function, and the 43 proteins were involved in 71 cellular functions. Specifically, within the reproduction-related group, 8 proteins were associated with fertilization or sperm-oocyte interaction (42%; SERPINA5, AKAP4, PRSS37, AKAP3, ROPN1B, SMCP, ELSPBP1, and GSG1), 7 with sperm motility and capacitation (37%; SEMG2, SEMG1, HYDIN, AKAP4, ROPN1B, SMCP, and ELSPBP1) and 4 with spermatogenesis (21%; SERPINA5, ROPN1B, SPATA19, SPANXB1) (**Figure 1**). Proteolysis and protein metabolism (15%), signaling (13%), immune system (11%), and metabolic process (8%) were the other prevailing cellular functions enriched for the sperm proteins with differential abundance in hypogonadism.

To unravel whether the deregulated sperm proteins in male hypogonadism were involved in explicit biological processes, we have performed GO term enrichment analysis using the Gene Ontology Consortium database based on PANTHER. The outcomes obtained confirmed that the sperm deregulated proteins in male hypogonadism were involved in fertilization, sperm capacitation, reproductive process and regulation of proteolysis (P-value and FDR adjusted P-value < 0.05). Concerning the GO Cellular Component, the deregulated sperm proteins were localized in the sperm fibrous sheath, acrosomal vesicle, extracellular exosome, extracellular matrix, and extracellular space (P-value and FDR adjusted P-value < 0.05).

### Western Blot Validation

**Figure 2:**
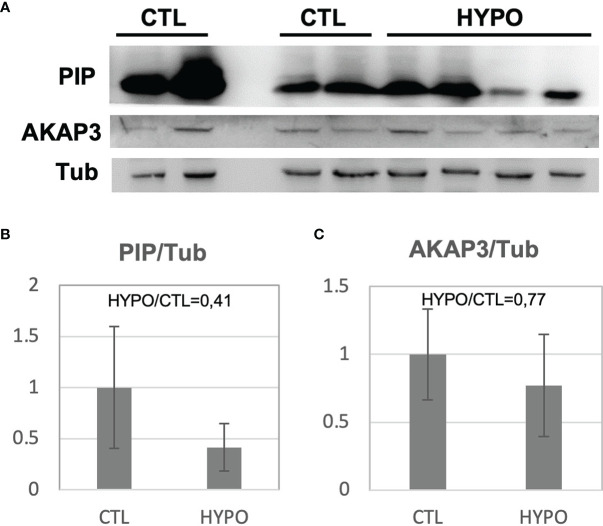
Western Blot analysis for the PIP and AKAP3 proteins obtained from sperm samples of patients with secondary hypogonadism (HYPO) and controls (CTL) **(A)**. The histogram shows the ratio of densitometric values of PIP **(B**) and AKAP3 **(C)** to Tubulin (Tub), a loading control. The ratio from control (CTL) was arbitrarily set to 1. Mean ± SD of patients is shown.

To validate the proteomic results, a Western blot analysis was performed for two of the proteins with differential abundance: A-Kinase Anchoring Protein 3 (AKAP3) and Prolactin Inducible Protein (PIP). Sperm protein extracts from 4 out of the 5 hypogonadic patients used for the proteomic analysis, and 4 out of the 5 fertile controls, were used for the Western blot analysis (two samples were completely used for proteomic analysis). As expected, significantly decreased levels (p<0.05) of PIP and AKAP3 were found in the secondary hypogonadic patients (**Figure 2A**), confirming the proteomics data. Indeed, the ratios for the 2 proteins among hypogonadics and controls, obtained from Western blot data (ratio 0.41 and 0.77, respectively), were very similar to the ones observed by proteomics data (ratio 0.51 and 0.74, respectively) (**Figures 2B, C**; **Table 2**).

## Discussion

The maintenance of spermatogenesis in humans requires an adequate secretion of LH, resulting in the maintenance of high intra-testicular testosterone (ITT) (2). In men and rats, the intratesticular T concentration is far higher than its concentration in the peripheral circulation (22). It is known that intratesticular T can be reduced substantially without an effect on spermatogenesis (5). However, there is a minimally required T concentration below which spermatogenesis is affected, leading to male infertility (23). T levels are approximately 40-fold higher in the testes than in the serum in healthy men with normal reproductive physiology. Noteworthy, it has been reported that T concentration in the testes in the absence of LH was still 4–5 times higher than serum T concentrations, since intratesticular T concentrations may still support spermatogenesis in men even if serum T is reduced (24).

In order to study if the reduction of serum T, although mild, might modify the qualitative protein composition of spermatozoa, we selected five patients affected by hypotestosteronemia due to isolated LH deficiency.

In previous studies, we reported the effect of male secondary post-surgical hypogonadism on modulating accessory gland function and protein secretion (25). In fact, in 2014 we published the first experimental proteomic study, using high-resolution mass spectrometry, aimed at studying the seminal proteome of patients affected by secondary hypogonadism, before and after 6 months of testosterone replacement treatment (26). More recently we reported a quantitative high-resolution proteomic research in seminal plasma samples of hypogonadotropic hypogonadism patients, before and after only 3 months of testosterone therapy (27), demonstrating the effect of male hypogonadism on modulating a panel of eleven seminal proteins.

In order to design adequate therapies for male infertility, it is essential to broaden our knowledge of the mechanisms underlying the role of testosterone in the initiation and maintenance of the poorly understood process of spermatogenesis, and in the pathogenesis of its disturbances.

Here, for the first time, we have quantified the changes occurring in the sperm proteome in patients with secondary hypogonadism, identifying 43 sperm proteins differentially expressed due to reduced serum T concentration. Thirty-two of these proteins were less abundant while 11 were more abundant in the hypogonadic patients.

Interestingly, many of these proteins have been previously reported to be involved in spermatogenesis, spermiation, sperm motility and capacitation, sperm-oocyte interaction, and/or fertilization. The reduction in hypogonadic patients of Prosaposin (PSAP), Ropporin-1B (ROPN1B), Plasma serine protease inhibitor (SERPINA5) and SPARC-related modular calcium-binding protein 1 (SMOC-1) might, in fact, reflect a dysregulation of the molecular machinery involved in spermiogenesis (28, 29), Sertoli cell junctions and blood-testis barrier (30, 31), spermiation (32), and epididymal shedding of cytoplasmic droplets (32–34). The increased abundance in hypogonadic patients of Sperm protein associated with the nucleus on the X chromosome B1 (SPANXB1) and Germ cell-specific gene 1 protein (GSG1) proteins, which have been previously reported as markers of defective spermatogenesis (35–37), and of Epididymal sperm-binding protein 1 (ELSPBP1) and fibronectin (FN1), which are considered markers of sperm damage and low sperm quality (38, 39), underlines the physiological role of testosterone in the qualitative control of sperm production.

The increase of 5-oxoprolinase (OPLAH) in hypogonadism might reflect a deficiency in the biosynthesis of glutathione, which is an important scavenger for reactive oxygen species in man (40). In addition, it is known that male hypogonadism is associated with oxidative stress, both at systemic (41) and at sperm level (42). The reported increase in OPLAH levels might be an indirect marker of oxidative stress in sperm of hypogonadic patients. We, therefore, further support the previously reported evidence in mice about the role of T in modulating the expression of testicular proteins involved in oxidative damage (11).

Moreover, we observed that male hypogonadism may impact sperm motility through the down-regulation of some specific proteins such as A-kinase anchor protein 3 (AKAP3), A-kinase anchor protein 4 (AKAP4), Hydrocephalus-inducing protein homolog (HYDIN) and ROPN1B, which are constitutive of the microtubule (43, 44) or necessary for the movement of the sperm tail (45–48).

Furthermore, ß-Microseminoprotein (MSMB) and Protein S100-A8 (S100A8) were reduced in sperm samples of hypogonadic patients. These proteins, although not annotated in UniProt Knowledgebase as involved in reproduction, have been previously reported in the literature as involved in preventing spontaneous acrosome reaction (49, 50).

Importantly, we reported in the panel of down-regulated proteins 9 seminal plasma proteins (Semenogelin-1 (SEMG1), Semenogelin-2 (SEMG2), myeloperoxidase (MPO), MSMB, SERPINA5, Prolactin-inducible protein (PIP), Prostate-specific antigen (KLK3), PSAP and Zinc-alpha-2-glycoprotein (AZGP1)). These proteins might derive from a residual quote of seminal plasma, might be attached to the cell membrane, or might be imported into the sperm cells through exosomes (51). However, it is interesting that we have previously reported in previous studies 8 out of 9 of these proteins (SEMG1, SEMG2, MPO, MSMB, PIP, KLK3, PSAP and AZGP1) to be downregulated in seminal plasma of patients with secondary male hypogonadism (26, 27). We, therefore, confirmed our previous data about the modulation of these seminal proteins in male secondary hypogonadism.

Overall, our data revealed the physiological role of intratesticular testosterone in modulating a molecular machinery directly involved in spermatogenesis and spermiogenesis, spermiation, sperm quality control, sperm motility and oxidative damage.

This *in vivo* model of intratesticular T deficit in humans allowed us to revisit the role of testosterone in spermatogenesis and in sperm function. Our findings suggest that Leydig cell dysfunction represents a mechanism responsible for the infertility of these patients. As a consequence, fertility can be effectively restored in these patients by hCG treatment. Thus, the identified proteins in this study might represent a target of responsiveness for hCG stimulation on sperm quality and fertility outcomes.

Furthermore, to date there is no approved serum biomarker for low intratesticular T. Intratesticular T can only be measured *via* invasive testicular biopsy or aspiration. We also speculate that the identification of these deregulated sperm proteins related to low intratesticular T may represent, if validated by further studies, putative non-invasive indirect biomarkers of intratesticular T.

## Conclusions

Despite the limitations of this study, performed on a small sample scale and pooled samples, this is the first application of high-resolution MS-based proteomics aimed to reveal an array of sperm proteins reflecting an impairment of spermatogenesis in testosterone deficiency. We performed our study in a clinical model – male hypogonadism due to isolated LH deficiency, in the presence of spermatogenesis – which is very rare. Thus, it is very difficult to enroll these patients. However, this rare clinical model permitted us to study spermatogenesis in presence of reduced T levels.

Further studies will be required to compare hypogonadic oligozoospermic samples with idiopathic oligozoospermic samples, in order to confirm the role of testosterone in the differentially expressed proteins.

The identification of a panel of proteins involved in androgen deficiency provides us a lesson on how androgens act under normal circumstances in the process of spermatogenesis and in the control of sperm function. Therefore, the identified proteins might represent new clinical biomarkers in androgen deficiency conditions.

## Data Availability Statement

The datasets presented in this study can be found in online repositories. The name of the repository and accession number can be found below: ProteomeXchange Consortium *via* the PRIDE partner repository (52); PXD032270.

## Ethics Statement

The studies involving human participants were reviewed and approved by Institutional Review Board of the International Scientific Institute “Paul VI”, Rome - Italy. The patients/participants provided their written informed consent to participate in this study.

## Author Contributions

GG, DM, FB, and RO contributed to conception and design of the study. GG, SC, and DM collected clinical data. GG, FB, AS-V, MJ, and FM performed formal analysis. GG, FB, and MJ performed data analysis. GG, FB, and DM wrote the first draft of the manuscript. RO and AP performed the supervision of the study. All authors contributed to manuscript revision, read, and approved the submitted version.

## Funding

This work was supported by the Spanish Ministry of Economy and Competitiveness (Ministerio de Economía y Competividad; fondos FEDER ‘una manera de hacer Europa’ PI16/00346 and PI20/00936 to RO), by the Spanish Ministry of Education, Culture and Sports (Ministerio de Educación, Cultura y Deporte para la Formación de Profesorado Universitario, FPU15/02306 to FB), and by the Government of Catalonia (Generalitat de Catalunya, pla estratègic de recerca i innovació en salut, PERIS 2016-2020, SLT002/16/00337 to MJ).

## Conflict of Interest

The authors declare that the research was conducted in the absence of any commercial or financial relationships that could be construed as a potential conflict of interest.

## Publisher’s Note

All claims expressed in this article are solely those of the authors and do not necessarily represent those of their affiliated organizations, or those of the publisher, the editors and the reviewers. Any product that may be evaluated in this article, or claim that may be made by its manufacturer, is not guaranteed or endorsed by the publisher.
